# Viral coinfection in hospitalized patients during the COVID-19 pandemic in Southern Brazil: a retrospective cohort study

**DOI:** 10.1186/s12931-024-02708-2

**Published:** 2024-02-05

**Authors:** Jaqueline Rhoden, Andressa Taíz Hoffmann, Janaína Franciele Stein, Bruna Seixas da Rocha, Vinícius Monteagudo de Barros, Eduardo Viegas da Silva, Juliane Deise Fleck, Caroline Rigotto

**Affiliations:** 1 Laboratório de Microbiologia Molecular, Universidade Feevale, Rodovia ERS-239, N◦ 2755, Prédio Vermelho, Piso 1, Sala 103, Vila Nova, Novo Hamburgo, Rio Grande Do Sul CEP 93525-075 Brazil; 2 Santa Casa de Misericórdia de Porto Alegre, Hospital Dom Vicente Scherer, Centro Histórico, Av. Independência, Nº 155, Porto Alegre, Rio Grande Do Sul CEP 90035- 074 Brazil; 3 Centro Estadual de Vigilância em Saúde do Rio Grande Do Sul, Av. Ipiranga, 5400, Jardim Botânico, Porto Alegre, Rio Grande Do Sul CEP 90450-190 Brazil

**Keywords:** SARS-CoV-2, Respiratory viruses, Infections, Vaccination

## Abstract

**Purpose:**

Since the worldwide spread of SARS-CoV-2, different strategies have been followed to combat the pandemic and limit virus transmission. In the meantime, other respiratory viruses continued to circulate, though at decreased rates.

**Methods:**

This study was conducted between June and July 2022, in a hospital in the metropolitan region of Rio Grande do Sul state, in the southernmost state of Brazil. The 337 hospitalized patients included those with respiratory symptoms without delimitation of age. Reverse transcription-quantitative real-time polymerase chain reaction detected 15 different respiratory viruses and confirmed coinfections in the samples. Different statistical tests were applied to evaluate the association between associations of clinical characteristics and coinfection.

**Results:**

Sampling corresponds to 337 selected and 330 patients analyzed. The principal clinical outcome found was hospital discharge in 309 (94%) cases, while 21 (6%) resulted in death. The principal viral agents related to coinfections were Human rhinovirus, Human enterovirus, and Respiratory syncytial virus. The most frequent viral agent detected was SARS-CoV-2, with 60 (18%) infections, followed by 51 (15%) cases of Respiratory syncytial virus B (15%) and 44 (13%) cases of Human rhinovirus 1. Coinfection was mainly observed in children, while adults and the elderly were more affected by a single infection. Analyzing COVID-19 vaccination, 175 (53%) were unvaccinated while the remainder had at least one dose of the vaccine.

**Conclusions:**

This study presents information to update the understanding of viral circulation in the region. Furthermore, the findings clarify the behavior of viral infections and possible coinfections in hospitalized patients, considering different ages and clinical profiles. In addition, this knowledge can help to monitor the population’s clinical manifestations and prevent future outbreaks of respiratory viruses.

**Supplementary Information:**

The online version contains supplementary material available at 10.1186/s12931-024-02708-2.

## Introduction

Infectious diseases are common, and their emergence reflects balances and imbalances among humans, animals, pathogens, and the environment. Respiratory tract infections can be caused by different viruses, and multiple factors contribute to progression and transmission [1]. One recent example is the severe acute respiratory syndrome coronavirus (SARS-CoV-2), which emerged from animal hosts and spread pandemically, causing coronavirus diseases 2019 (COVID-19) [2, 3].

Countries developed different strategies to combat the outbreak, like maintaining physical distance and wearing face masks. With these restrictions on public life, other viral agents were also impacted through reduced circulation [4–6]. Moreover, respiratory viruses are affected by seasonal conditions and commonly present different detection rates along the year [7, 8].

The gravity and clinical characteristics of respiratory viruses are mainly related to patients' susceptibility aggravated by comorbidities and immunosuppression [1, 9]. Clinical symptoms associated with different viruses causing illnesses overlap and are often indistinguishable, affecting patients of all ages [10]. Pediatric patients are mainly affected by respiratory infections which are one of the major causes of morbidity and mortality [11, 12].

As such, this study investigates insights into viral circulation and coinfections relationship with clinical characteristics and vaccination status in hospitalized patients in a hospital in the southernmost state of Brazil.

## Methods

### Ethical approval

This study was approved by the Research Ethics Committee at Santa Casa de Misericórdia de Porto Alegre Hospital, according to National Commission for Research Ethics. File number: 57888422.3.0000.5335—Ethical Review Presentation Certificate.

### Study design and participants

This retrospective cohort study included 337 patients admitted to a hospital in June and July 2022, which were the coldest months of the winter season. The hospital is situated in the city of Porto Alegre, the capital of Rio Grande do Sul, the southernmost state of Brazil. Study participants were patients with respiratory symptoms and hospitalized, some of whom were in the intensive care unit (ICU), irrespective of positive or negative COVID-19 confirmation test (RT-qPCR). Those who took the test and were not hospitalized were excluded. Without delimitation of age, the sampling was classified into age groups: infants (0–4 years), children (5–12 years), adults (13–59), and elderly (> 60).

### Data collection

The clinical and epidemiological characteristics and hospitalization outcomes (use of supplemental oxygen and invasive mechanical ventilation, admission to ICU, and death or discharge) were collected in medical records (Tasy 3.07.1815.133 Software) for each patient and accessed directly at the hospital.

The vaccination data were collected on the national immunization program. The classification of COVID-19 vaccine status was: fully vaccinated, partially vaccinated, or unvaccinated, and defined based on the number of doses by age, according to the immunization panel standardized by health normative from the Brazilian Government [13]. During the period of the study, fully vaccinated patients were defined as those over 40 years old with 4 doses, adults between 12 and 39 years old with 3 doses, children aged between 5 and 11 years old with 2 doses, and children (infants) under 4 years old and not recipients of the COVID-19 vaccine. Those without the respective number of doses were classified as partially vaccinated. Vaccination data for other viruses such as Influenza were not collected due to a lack of information in the medical records.

### Viral detection

Naso-oropharyngeal swabs, tracheal and nasopharyngeal aspirate, and sputum samples were obtained from the molecular biology laboratory in the hospital and sent to the Laboratório de Microbiologia Molecular—Feevale University. Initially, nucleic acid extraction was performed using the MagMAX™ CORE Nucleic Acid Purification Kit (Applied biosystems™, Thermo Fisher Scientific, Waltham, MA, USA) with automated KingFisher™ Duo Prime (Thermo Fisher Scientific™) equipment. Reverse transcription-quantitative real-time polymerase chain reaction (RT-qPCR) was performed using specific assays (Thermo Fisher Scientific, Waltham, MA, USA) with probe and primers to detect human adenovirus type C (HAdV); human bocavirus (HBoV); coronavirus 229E (CoV 229E); human enterovirus (EV); metapneumovirus (hMPV); parainfluenza virus types 1, 2, 3 and 4 (HPIV); influenza A virus types H1 and H3 (FLUAV); influenza B virus (FLUBV); respiratory syncytial virus (RSV) types A and B; and human rhinovirus types A and B (HRV).

SARS-CoV-2 detection by RT-qPCR was performed using the *Charite* Institute protocols (Berlin, Germany) that detects the envelope (E) gene. RT-qPCR was performed with a TaqMan^®^ Microbial Assays-single tube assay (Applied Biosystems, Pleasanton, California, USA). The probe access codes for the target pathogens are listed in Additional file 1: Table S1.

RNA virus detection employed AgPath-ID™ One-Step RT-PCR Master Mix (4,387,391, Applied Biosystems). TaqPath™One-Step RT-qPCR Master Mix CG (A15299, Applied Biosystems) and TaqMan™ Fast Advanced Master Mix (4,444,557, Applied Biosystems) reagents were used to detect the DNA virus. Taq-Man^®^Respiratory Tract Microbiota Amplification Control (A39178, Thermofisher) was used for reaction control. Detections with Cycle Threshold (Ct) up to 34 were considered positive, in conformity with manufacturer recommendations.

For data exploration, viral detection and coinfection were classified into four patient groups: negative; one virus; two viruses; and three or more viruses.

### Statistical analysis

The distribution of variables used in the analyses was initially investigated in terms of absolute and relative values for each category. Afterward, bivariate analyses—crude association with outcomes –, were performed using the Chi-square test. Subsequently, considering age as a potentially strong confounder, those bivariate analyses were stratified by four age range groups (0–4; 5–12; 13–59; > 60 years), and the results are presented in the supplemental materials.

Multivariable logistic regression analyses were performed to evaluate associations between single respiratory viruses infections (1 virus) and coinfections (2, 3 or more viruses detected in the same patient), and dichotomous outcomes including symptoms (coughing, dyspnea, fever, coryza, vomiting, sore throat, nausea, diarrhea, headache, mental confusion), characteristics of the hospitalization (ICU, supplemental oxygen, and mechanical ventilation) and outcome (death or hospital discharge). Linear regression was used for the length of hospitalization in days. For all multivariable models, a set of potential confounders were considered, related to demographic characteristics (age and sex), comorbidities (hypertension, smoking, cardiovascular disease, neoplasm, diabetes, asthma, neurological disease, obesity, COPD, dyslipidemia, transplants, kidney disease, others), and the COVID-19 vaccination status. Starting from this initial “full” model, a backward selection process was carried out, maintaining confounders at a p-value < 0.20. To confirm that no relevant confounders were mistakenly excluded, each covariate excluded throughout the backward selection process was reintroduced in the final model and finally removed if weakly associated (p-value < 0.20) with the outcomes. Finally, after achieving the final model, the statistical significance of the association between the exposure of interest and each outcome was examined using the likelihood ratio test. All data preprocessing and analyses were performed using Stata 15.0 statistical software.

## Results

Of the 337 screened patients, 7 were excluded (for failure in data collection). Hence, the complete case analysis included 330 individuals. Of these, 172 (52%) were male patients, and 158 (48%) were female. Admission occurred in all departments of the hospital; 190 (58%) patients corresponded to the pediatric department. The age range varied from 5 days to 97 years old; the average age was 28 years old. Average admission time was 15 days; and 309 (94%) patients were discharged from hospital, with 21 (6%) deaths recorded. The cause of death was not described in the medical records. Of the total number of cases, 219 (66%) presents at least one viral infection. Clinical features and viral coinfections are presented in Table 1.

**Table 1:** Patients’ clinical characteristics according to the number of viral agents detected and coinfection (n = 330)

Characteristics	Viral detection				n	p value
	0	1	2	≥ 3		
Sex						**0.034**
Male (M)	62 (36%)	63 (37%)	33 (19%)	14 (8%)	172	
Female (F)	49 (31%)	81 (51%)	17 (11%)	11 (7%)	158	
Age group (years)						**0.000**
Infants (0–4)	31 (21%)	61 (40%)	39 (26%)	20 (13%)	151	
Children (5–12)	14 (36%)	17 (44%)	4 (10%)	4 (10%)	39	
Adults (13–59)	27 (51%)	22 (41%)	4 (8%)	0 (0%)	53	
Elderly (> 60)	39 (45%)	44 (51%)	3 (3%)	1 (1%)	87	
Average age	28 years					
Comorbidities						
Hypertension	31 (42%)	38 (51%)	4 (6%)	1 (1%)	74	**0.003**
Smoker	26 (47%)	25 (46%)	4 (7%)	0 (0%)	55	**0.010**
Cardiovascular disease	25 (49%)	24 (47%)	2 (4%)	0 (0%)	51	**0.003**
Neoplasm	25 (53%)	19 (40%)	3 (7%)	0 (0%)	47	**0.004**
Diabetes	20 (55%)	14 (39%)	1 (3%)	1 (3%)	36	**0.010**
Asthma	12 (38%)	11 (34%)	3 (9%)	6 (19%)	32	0.058
Neurological disease	4 (21%)	14 (74%)	1 (5%)	0 (0%)	19	**0.047**
Obesity	9 (53%)	7 (41%)	1 (6%)	0 (0%)	17	0.223
COPD	9 (53%)	8 (47%)	0 (0%)	0 (0%)	17	0.106
Dyslipidemia	6 (40%)	8 (53%)	0 (0%)	1 (7%)	15	0.403
Transplants	5 (38%)	7 (54%)	1 (8%)	0 (0%)	13	0.585
Kidney disease	4 (36%)	6 (55%)	1 (9%)	0 (0%)	11	0.688
Others^a^	47 (38%)	60 (48%)	14 (11%)	4 (3%)	125	**0.030**
Symptomatologic						
Cough	73 (30%)	105 (44%)	40 (17%)	22 (9%)	240	0.072
Dyspnea	61 (33%)	74 (41%)	28 (15%)	20 (11%)	183	0.069
Fever	44 (27%)	74 (45%)	34 (21%)	11 (7%)	163	**0.009**
Coryza	30 (24%)	55 (43%)	29 (23%)	13 (10%)	127	**0.001**
Vomit	16 (30%)	21 (40%)	11 (21%)	5 (9%)	53	0.562
Sore throat	9 (41%)	9 (41%)	3 (14%)	1 (4%)	22	0.866
Nausea	6 (29%)	12 (57%)	3 (14%)	0 (0%)	21	0.420
Diarrhea	3 (19%)	8 (50%)	4 (25%)	1 (6%)	16	0.497
Headache	7 (50%)	6 (43%)	0 (0%)	1 (7%)	14	0.336
Mental confusion	5 (42%)	7 (58%)	0 (0%)	0 (0%)	12	0.298
Admission						
Supplemental oxygen	60 (32%)	78 (41%)	33 (17%)	20 (10%)	191	**0.049**
ICU	17 (35%)	21 (44%)	7 (15%)	3 (6%)	48	0.978
Mechanical ventilation	12 (35%)	17 (50%)	2 (6%)	3 (9%)	34	0.456
Hospitalization time (days)						**0.005**
≤ 5	28 (23%)	55 (45%)	26 (21%)	14 (11%)	123	
06 to 10	25 (33%)	31 (41%)	13 (17%)	7 (9%)	76	
11 to 20	24 (43%)	23 (41%)	6 (11%)	3 (5%)	56	
≥ 21	34 (45%)	35 (47%)	5 (7%)	1 (1%)	75	
Average time of admission	15 days					
Clinical outcome						0.324
Hospital discharge	101 (33%)	135 (44%)	48 (15%)	25 (8%)	309	
Death	10 (48%)	9 (43%)	2 (9%)	0 (0%)	21	
COVID—19 vaccination						**0.000**
No vaccination	38 (22%)	73 (42%)	40 (23%)	24 (13%)	175	
Partially	39 (46%)	40 (47%)	6 (7%)	0 (0%)	85	
Complete	34 (49%)	31 (44%)	4 (6%)	1 (1%)	70	
Total	111 (34%)	144 (44%)	50 (15%)	25 (7%)	330	

^a^Other comorbidities include hypothyroidism, benign prostatic hyperplasia, cirrhosis, gastroesophageal reflux, Down syndrome, epilepsy, psychiatric disorders (depression, anxiety, panic disorder, and autism), infectious diseases (HIV, hepatitis B, hepatitis C, and syphilis)

p-value for heterogeneity Chi-square test. Significant values are highlighted in bold

### Clinical characteristics

According to the medical records, the most common symptoms reported were coughing 240 (73%), dyspnea 183 (55%), fever 163 (49%), and coryza 127 (38%). In comorbidities, hypertension was presented in 74 (22%) cases as the most frequent disease. In turn, children who were generally free of comorbidities represent more than half of the sample of this study.

Evaluating average hospitalization time, 123 (37%) patients spent up to 5 days in the hospital, and 75 (23%) spent 21 or more days, resulting in an average hospitalization time of 15 days. Supplemental oxygen was used in 191 (58%) cases, and of these 42 (22%) evolved to ICU admission. In total, 48 (15%) patients were admitted to the ICU, and 13 (27%) deaths were recorded, with significant association (p < 0.05) between the gravity of cases and death.

### Vaccination

**Fig. 1:**
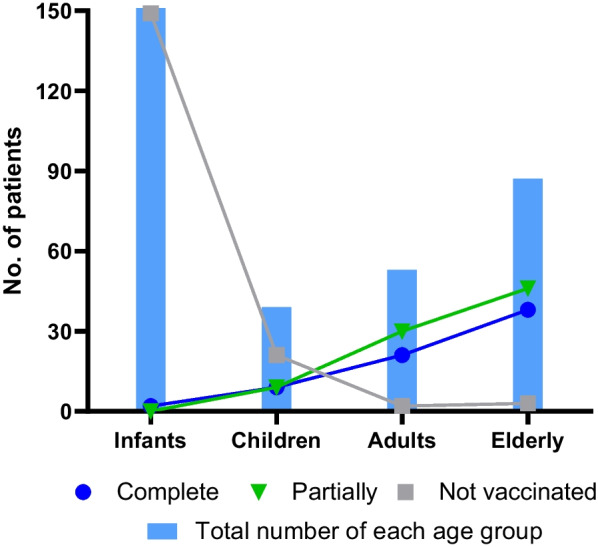
Vaccination status divided by number of patients from each age group

Considering COVID-19 vaccination in Brazil, the available vaccines at the time of the study were CoronaVac (Sinovac Biotech), ChAdOx1 nCov-19 (AstraZeneca/Oxford University), BNT162b2 (Pfizer–BioNTech), and Ad26.Cov2.S (Johnson & Johnson–Janssen). In this study, 155 (47%) patients were vaccinated with at least 1 dose, and 175 (53%) were unvaccinated. Regarding the vaccination immunization panel for COVID-19, 85 (25%) patients corresponded to a status of partial vaccination, while 70 (21%) held the status of fully vaccinated. Analyzing each age group and respective status (Fig. 1), 149 (85%) of the patients were infants and unvaccinated. This is an expected result since these age ranges were not included on the immunization panel during the study period. Of the partially vaccinated, 46 (54%) were elderly, and 30 (35%) were adults. In the fully vaccinated group, 38 (54%) were elderly, 21 (30%) were adults, 9 (13%) were children and 2 (3%) were infants. One of the 2 infant patients had bone neoplasm, and the other suffered from comorbidities, which explains the reason for vaccination.

**Fig. 2:**
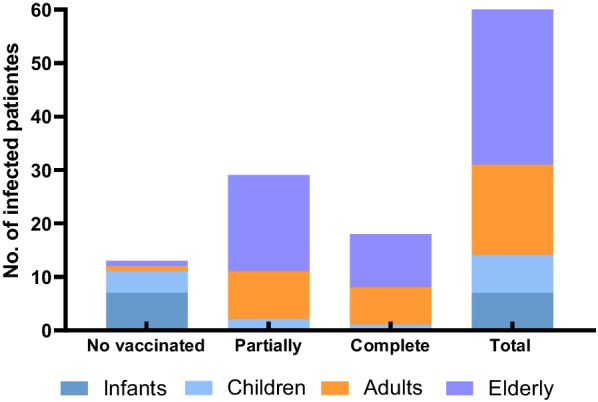
Vaccination status of SARS-CoV-2 infected divided by age group (n = 60)

Analyzing the vaccination status and patients infected by SARS-CoV-2 (n = 60), 13 (22%) were unvaccinated, 29 (48%) were partially, and 18 (30%) were fully vaccinated (Fig. 2). Of the 21 deaths, 5 (24%) were infected by SARS-CoV-2. Of these, 3 patients were partially vaccinated, 1 was unvaccinated and 1 was fully vaccinated. Results presented an association (p < 0.05) between SARS-CoV-2 infection and incomplete vaccination status.

### Viral detection

**Fig. 3:**
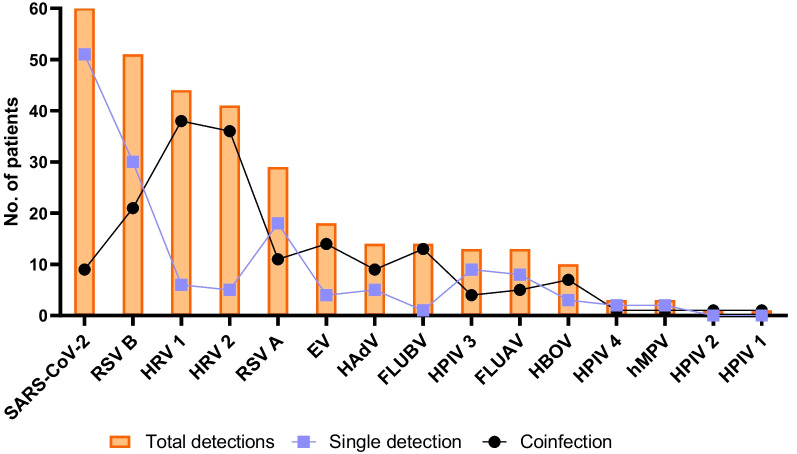
Total number of patients with each viral detection and the distribution of single or coinfection

Viral detection evaluated 15 viruses and among them, only CoV 229E was not detected. Our study showed different rates of detection among the viral agents when analyzing single or coinfection (Fig. 3). In total, 219 (66%) patients were infected with at least one virus. Of these, 144 (66%) presented single infections, 50 (23%) were coinfected by 2 viruses, and 25 (11%) by 3 or more viruses. In total, 75 patients were coinfected. Our analyses revealed that coinfection was significantly associated with age, with younger people (infants and children) presenting more coinfections than adults and elderly patients (79%, 11% vs. 5%, 5%, p = 0.000).

**Fig. 4:**
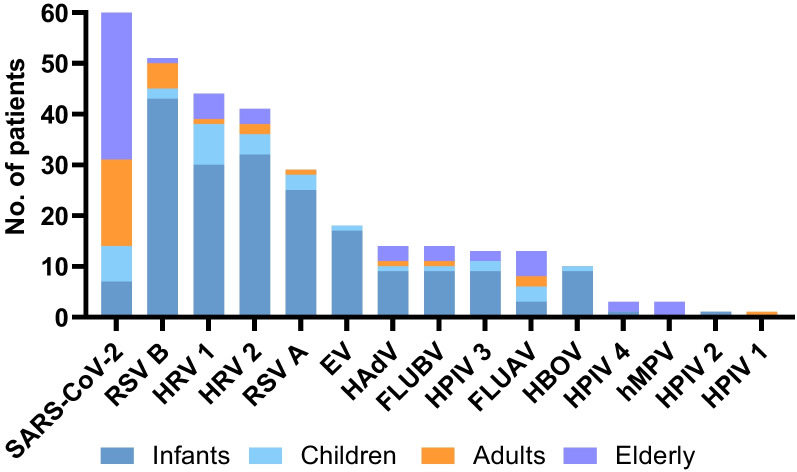
Distribution of viral infection by age groups

When each virus was analyzed separately, the most frequent was SARS-CoV- 2, with 60 (18%) infections, followed by 51 (15%) RSV B, 44 (13%) HRV 1, 41 (12%) HRV 2, and 29 (9%) RSV A. Analyzing by age groups (Fig. 4), RSV B and RSV A where the most frequently detected in infants, while SARS-CoV-2, FLUAV and RV1 were more common among the elderly. Children and adults showed minor detection rates.

For single detection, SARS-CoV-2 was responsible for 51 (35%) infections, followed by 30 (21%) with RSV B and 18 (13%) with RSV A. For coinfection with 2 viruses, HRV 1 + HRV 2 were detected in 8 (16%) patients, HRV 1 + EV in 6 (12%), and RSV A + B in 5 (10%). Moreover, the main agents associated with 3 or more coinfections were HRV 1 + HRV 2 + EV, observed in 10 (40%) patients. Followed by HRV 1 + HRV 2 + EV + HAdV, and HRV 1 + HRV 2 + EV + RSV B, detected in 3 (12%) and 2 (8%) patients, respectively. The total number of detections and frequency are shown in the supplementary material (Additional file 2**:** Table S2).

In unadjusted analysis, coinfections were significantly associated (p < 0.05) with age, sex, hospitalization time, comorbidities (hypertension, smoking, cardiovascular disease, neoplasm, diabetes, COPD, and others), symptoms (coughing, fever, and coryza), and use of supplemental oxygen. These results are shown in Table 1.

In adjusted analysis, fever was associated in cases with 2 coinfections (adjusted odds ratio [aOR], 2.248; CI 1.057–4.780) while dyspnea was associated with 3 or more (aOR, 3.983; 1.324–11.975). Upon evaluating the presence of any type of coinfection (no/yes), dyspnea (aOR, 1.938; CI 1.084–3.464) and coryza (aOR, 1.804; CI 1.016–3.203) showed association. Variables associated with SARS-CoV-2 infections were dyspnea (aOR, 0.508; CI 0.269–0.956) and coughing (aOR, 0.449; CI 0.240–0.840). A complete adjusted analysis between coinfections and hospitalization outcomes is shown in the supplemental material (Additional file 3**:** Table S3).

## Discussion

This study divided the data into specific groups for suitable analysis. Generally, symptoms associated with respiratory infections are unspecific, however, our study showed that dyspnea was reported in each kind of evaluated infection, considering single, double or triple infection by viral agents [14]. This was an expected result since this symptom is relevant for hospitalizations due to respiratory infections. Fever and coryza also were associated with coinfection while coughing was linked to SARS-CoV-2. This finding may suggest that existing symptoms are related to the kind of virus coinfections but cause similar clinical features.

According to other studies, fever and coughing were the most common symptoms in children since pediatric fever is often associated with infection, and viral infections are a considerable cause [15]. Among the elderly, dyspnea is more common and represents a cause of reduced pulmonary gas exchanges; consequently, the group is more likely to present respiratory issues and hypoxemia. With cardiovascular comorbidity or associated respiratory disease, this symptom can be a considerable aggravating factor [16].

Diabetes, cardiovascular and pulmonary diseases are often associated with an increased fatality rate in respiratory disease [17]. Like other studies, a prevalence of hypertension was also noted in the sample group, with age and obesity also important risk factors linked to hospital admission [17]. Since children are less prone to diseases like diabetes, hypertension, or other cardiovascular disease, this portion of the population had a lower risk of developing severe infectious conditions. This data can explain the higher number of severe cases reported among the elderly since they ordinarily suffer from more associated comorbidities [15, 18].

Studies reported that mortality is associated with ICU and even more so with nosocomial infection [19, 20]. Many cases require hospitalization, and with preexisting diseases, the patients have a greater possibility of coinfections, mainly RSV and FLUAV [14, 21, 22]. Similar studies showed an increased risk of death associated with coinfection [6]. In this study, ICU and death did not present the same association though there was a relation (p < 0.05) between the use of supplemental oxygen and coinfection, emphasizing that it compromises the respiratory system.

The prevalence of SARS-CoV-2 and other respiratory viruses varied throughout the COVID-19 pandemic waves with declines in FLUAV and FLUBV, RSV, HRV, and EV infections, which are the most commonly described [6, 17, 23]. Other viruses such as HAdV, HPIV, and hMPV were less detected compared to previous years, even during the winter season when these viruses commonly present higher rates of detection [17, 22, 24]. HRV was the main pathogen to substantially cocirculate with SARS-CoV-2 during the pandemic period and remains present at a considerable rate (~ 20%) [5, 17, 25, 26].

The most frequent viruses related to coinfection were HRV and EV, like the results of this study [22, 23]. Belonging to the viral family *Picornaviridae*, different viral subtypes are associated with respiratory infections, mainly HRV1, HRV2 and EV-D68 [22, 23]. The inherent structure and properties of these viruses and the absence of the viral envelope confer persistence and resistance to environmental factors [23]. Consequently, even under seasonal conditions, their continued circulation has been reported [7, 23]. HRV and RSV were common pathogens that typically cause upper respiratory tract disease and mainly affect children, causing pneumonia and bronchiolitis [8, 22, 26]. According to another researcher, this study’s findings also presented higher rates of RSV and most likely did not show increased rates of preventive measures for COVID-19. The clinical presentations of these 2 infections were generally similar, but RSV is more related to severe cases and outcomes [8]. In this condition, RSV is the leading cause of morbidity and mortality in younger children [8, 26].

According to our analyses, the results with the association between coinfection and younger people were consistent with other studies examining coinfections in similar sampling groups [6, 22]. One acceptable explanation is the immature immune system in the youngest patients [6]. Moreover, respiratory viral infections involve complex interactions when a host is infected. Mechanisms of viral interference an innate immune response may be stimulated, and the interferon release may inhibit other respiratory viral infections [23].

The vaccination measures to control the pandemic resulted in fewer cases of infection, reducing the severity outcomes [17]. However, inequality in vaccine distribution was noted. The extent of primary vaccination and booster coverage in our study sample was higher than in earlier periods of the pandemic, and children’s vaccination was still at an initial phase [25]. The high rate of unvaccinated patients found in this study is explained by the number of infants and children, who were not included on the national vaccination panel during the study period.

In Brazil, vaccination against COVID-19 began in January 2021 [13]. At the time this study was carried out, the population was already largely vaccinated, which can explain the 5 (8%) deaths from total SARS-CoV-2 cases. COVID-19 cases were related to ICU admission and death among the elderly, so hospitalizations rose rapidly after the increase in cases and with the high transmissibility of the Omicron variant, responsible for the COVID-19 wave [27]. In this study, the elderly were the main fully vaccinated age group, as expected.

The vaccine effectiveness against severe hospitalization cases and death conferred protection in the primary series of vaccinations, with immunity waning over time [28–30]. With the onset of the Omicron wave, the recommendation of booster shots provided greater protection, mainly during hospitalization of the elderly [28]. Studies suggest that heterologous booster administration afforded a substantial increase in protection [30, 31]. Therefore, they significantly helped mitigate worse outcomes [29, 31].

Throughout the COVID-19 pandemic, the concomitant circulation of viral agents was discussed, along with the difficulty of accurately estimating this circulation. However, with decreasing detection rates for the most prevalent respiratory viruses, these comparisons were based on retrospective data. Coinfection was mainly observed in children, while adults and the elderly were more affected by a single infection.

This study has some limitations. First, the lack of access to the cause of death prevented us from concluding whether the infection was an exacerbating factor or the cause. Thus, severity assessment introduces bias. Secondly, we did not differentiate the subtypes of EV infection as we lacked specific assays for each subtype. However, this study offers data to update the epidemiological situation in the region and clarifies the impact of coinfections together with clinical analysis of the conditions presented by patients. Understanding the association of different factors, such as seasonality and distribution of age groups affected by respiratory viruses, can contribute to better prevention measures, while also minimizing consequences for patients and public health.

### Supplementary Information

**Additional file 1: Table S1.** Probe access code for the target pathogens used in the RT-qPCR.

**Additional file 2: Table S2.** Total number of detections and frequency from each virus evaluating total number of patients (n = 330), single infections (n = 144), 2 viruses coinfected (n = 50) and 3 or more coinfected (n = 25).

**Additional file 3: Table S3.** Associations between coinfections and hospitalization outcomes—Crude and adjusted Odd ratios (OR) (n = 330).

## Data Availability

The dataset supporting the conclusions of this article are included within the article and its additional files.
