# Supplementary material for: Viral coinfection in hospitalized patients during the COVID-19 pandemic in Southern Brazil: a retrospective cohort study
Source: Respir Res. 2024 Feb 5;25:71. doi: 10.1186/s12931-024-02708-2 (PMC10840208; doi:10.1186/s12931-024-02708-2)
Supplement: Supplementary file 1 — Additional file 1: Table S1. Probe access code for the target pathogens used in the RT-qPCR. [file 12931_2024_2708_MOESM1_ESM.docx]

**Table S1: Probe access code for the target pathogens used in the RT-qPCR**

| Target | Sequencies / Assay ID | Reference |
| --- | --- | --- |
| Human adenovirus type C | VTB2-HAdVCf - GAGACGTACTTCAGCCTGAAT | (Wolf, 2010) |
|  | VTB2-HAdVCr - GATGAACCGCAGCGTCAA |  |
|  | VTB2-HAdVCprobe - CCTACGCACGACGTGACCACAGA |  |
| Bocavirus | Vi99990003_po | *ThermoFisher Scientific* |
| Coronavirus 229E | Vi06439671_s1 | *ThermoFisher Scientific* |
| SARS-COV-2 - gene E | E -Sarbeco-F ACAGGTACGTTAATAGTTAATAGCGT | (Charité, 2020) |
|  | E_Sarbeco_R ATATTGCAGCAGTACGCACACA |  |
|  | E_Sarbeco_P FAM ACACTAGCCATCCTTACTGCGCTTCGBBQ |  |
| Human Enterovirus - PAN | Vi06439631_s1 | *ThermoFisher Scientific* |
| Influenza A - H1 | Vi99990009_po | *ThermoFisher Scientific* |
| Influenza B - PAN | Vi06439668_s1 | *ThermoFisher Scientific* |
| Metapneumovírus | Vi99990004_po | *ThermoFisher Scientific* |
| Parainfluenza 1 | Vi06439642_s1 | *ThermoFisher Scientific* |
| Parainfluenza 2 | Vi06439672_s1 | *ThermoFisher Scientific* |
| Parainfluenza 3 | Vi06439670_s1 | *ThermoFisher Scientific* |
| Parainfluenza 4 | Vi99990005_po | *ThermoFisher Scientific* |
| Respiratory Syncytial Virus A | Vi99990014 | *ThermoFisher Scientific* |
| Respiratory Syncytial Virus B | Vi99990015 | *ThermoFisher Scientific* |
| Rhinovirus 1 | Vi99990016_po | *ThermoFisher Scientific* |
| Rhinovirus 2 | Vi99990017_po | *ThermoFisher Scientific* |

WOLF, S. Viral multiplex quantitative PCR assays for tracking sources of fecal contamination. Applied and Environmental Microbiology, v. 76, n. 5, p. 1388–1394, 2010.

CORMAN, V. et al. Diagnostic detection of 2019-nCoV by real-time RT-RCR. Charité Berlin, p. 13, 2020.
