# Supplementary material for: Viral coinfection in hospitalized patients during the COVID-19 pandemic in Southern Brazil: a retrospective cohort study
Source: Respir Res. 2024 Feb 5;25:71. doi: 10.1186/s12931-024-02708-2 (PMC10840208; doi:10.1186/s12931-024-02708-2)
Supplement: Supplementary file 2 — Additional file 2: Table S2. Total number of detections and frequency from each virus evaluating total number of patients (n = 330), single infections (n = 144), 2 viruses coinfected (n = 50) and 3 or more coinfected (n = 25). [file 12931_2024_2708_MOESM2_ESM.docx]

**Table S2: total number of detections and frequency from each virus evaluating total number of patients (n=330), single infections (n = 144), 2 viruses coinfected ( n= 50) and 3 or more coinfected ( n= 25).**

| Respiratory virus | n | % |
| --- | --- | --- |
|  | 330 |  |
| SARS-CoV-2 | 60 | 18 |
| Respiratory syncytial virus B | 51 | 15 |
| Human rhinovirus 1 | 44 | 13 |
| Human rhinovirus 2 | 41 | 12 |
| Respiratory syncytial virus A | 29 | 9 |
| Human enterovirus | 24 | 7 |
| Human adenovirus | 18 | 5 |
| Influenza B virus | 14 | 4 |
| Parainfluenza virus 3 | 13 | 4 |
| Influenza A virus | 13 | 4 |
| Human bocavirus | 10 | 3 |
| Parainfluenza virus 4 | 3 | 1 |
| Human metapneumovirus | 3 | 1 |
| Parainfluenza virus 2 | 1 | 1 |
| Parainfluenza virus 1 | 1 | 1 |
|  | | |
| Single infection | 144 | 66 |
|  | | |
| SARS-CoV-2 | 51 | 35 |
| Respiratory syncytial virus B | 30 | 21 |
| Respiratory syncytial virus A | 18 | 13 |
| Parainfluenza virus 3 | 9 | 6 |
| Influenza A virus | 8 | 6 |
| Others^a^ | 28 | 19 |
|  | | |
| Coinfection |  |  |
| 2 agents | 50 | 23 |
|  | | |
| Human rhinovirus (1 and 2) | 8 | 16 |
| Human rhinovirus 1 + Enterovirus | 6 | 12 |
| Respiratory syncytial virus (A and B) | 5 | 10 |
| Others^b^ | 31 | 62 |
|  | | |
| 3 or more | 25 | 11 |
|  | | |
| Human rhinovirus (1 and 2) + Enterovirus | 10 | 40 |
| Human rhinovirus (1 and 2) + Enterovirus + Human adenovirus | 3 | 12 |
| Human rhinovirus (1 and 2) + Human adenovirus | 2 | 8 |
| Human rhinovirus (1 and 2) + Enterovirus + Respiratory syncytial virus B | 2 | 8 |
| Others^c^ | 8 | 32 |
|  |  |  |
| No detection | 111 | 34 |

^a^ Human rhinovirus 1; Human rhinovirus 2; Influenza B; Human adenovirus; Human bocavirus; Metapneumovirus; Parainfluenza virus 4; Human enterovirus.

^b^ Respiratory syncytial virus B + Influenza B; Respiratory syncytial virus B + Human adenovirus; Respiratory syncytial virus A + Human bocavirus; Respiratory syncytial virus B + Human bocavirus; Respiratory syncytial virus A + Human rhinovirus 2; Respiratory syncytial virus B + Influenza A; Human rhinovirus 2 + Human adenovirus; Human rhinovirus 2 + Influenza B; Human rhinovirus 1 + Influenza B; Human rhinovirus 2 + Human bocavirus; Respiratory syncytial virus A + Parainfluenza virus 2; SARS-CoV-2 + Human rhinovirus 2; SARS-CoV-2 + Respiratory syncytial virus A; SARS-CoV-2 + Respiratory syncytial virus B; SARS-CoV-2 + Influenza A; SARS-CoV-2 + Influenza B; SARS-CoV-2 + Parainfluenza virus 1; SARS-CoV-2 + Parainfluenza virus 3; Parainfluenza virus 3 + Human bocavirus; Parainfluenza virus 3 + Human rhinovirus 2; Parainfluenza virus 4 + Human adenovirus; Influenza A + Human adenovirus.

^C^ Human rhinovirus (1 and 2) + Human adenovirus; Human rhinovirus (1 and 2) + Respiratory syncytial virus B; Human rhinovirus (1 and 2) + Metapneumovirus; Human rhinovirus (1 and 2) + Influenza B; Human rhinovirus (1 and 2) + Human bocavirus; Respiratory syncytial virus B + Influenza B + Human adenovirus; SARS-CoV-2 + Human enterovirus + Human rhinovirus 1; Human enterovirus + Human rhinovirus 1 + Human adenovirus; Human rhinovirus 2 + Influenza A, Human bocavirus + Respiratory syncytial virus B.
