# Supplementary material for: Viral coinfection in hospitalized patients during the COVID-19 pandemic in Southern Brazil: a retrospective cohort study
Source: Respir Res. 2024 Feb 5;25:71. doi: 10.1186/s12931-024-02708-2 (PMC10840208; doi:10.1186/s12931-024-02708-2)
Supplement: Supplementary file 3 — Additional file 3: Table S3. Associations between coinfections and hospitalization outcomes—Crude and adjusted Odd ratios (OR) (n = 330). [file 12931_2024_2708_MOESM3_ESM.docx]

**Table S3**: **Associations between coinfections and hospitalization outcomes – Crude and adjusted Odd ratios (OR) (n = 330)**

|  | | **Characteristic /outcome** | | | |
| --- | --- | --- | --- | --- | --- |
| **Exposure** | **(p-value)** | **Crude OR** | **95% IC** | **Adjusted OR^a^** | **95% IC** |
|  |  | Mental confusion | | | |
| Number of virus infection/coinfection | **-** | - | - | - | - |
| 1 |  | - | - | - | - |
| 2 |  | - | - | - | - |
| 3 |  | - | - | - | - |
| Coinfection | **-** |  |  |  |  |
| Yes / No |  |  |  |  |  |
| SARS-CoV-2 detection | 0.561 |  |  |  |  |
| Yes / No |  | 4.888 | 1.519 - 15.733 | 1.485 | 0.393 - 5.606 |
|  |  |  |  |  |  |
|  |  | Fever | | | |
| Number of virus infection/coinfection | **0.040ǂ** |  |  |  |  |
| 1 |  | 1.609 | 0.974 - 2.657 | 1.467 | 0.866 - 2.484 |
| 2 |  | **3.235** | **1.598 - 6.551** | **2.248** | **1.057 - 4.780** |
| 3 |  | 1.196 | 0.497 - 2.874 | 0.651 | 0.256 - 1.656 |
| Coinfection | 0.557ǂ |  |  |  |  |
| Yes / No |  | 1.441 | 1.031 - 2.939 | 1.187 | 0.668 - 2.108 |
| SARS-CoV-2 detection | 0.468ǂ |  |  |  |  |
| Yes / No |  | 0.742 | 0.422 - 1.304 | 1.262 | 0.672 - 2.371 |
|  |  |  |  |  |  |
|  |  | Dyspnea | | | |
| Number of virus infection/coinfection | **0.044ǂ** |  |  |  |  |
| 1 |  | 0.866 | 0.527 - 1.423 | 1.027 | 0.606 - 1.742 |
| 2 |  | 1.043 | 0.532 - 2.042 | 1.508 | 0.731 - 3.110 |
| 3 |  | **3.278** | **1.148 - 9.358** | **3.983** | **1.324 - 11.975** |
| Coinfection | **0.023ǂ** |  |  |  |  |
| Yes/ No |  | 1.580 | 0.928 - 2.689 | **1.938** | **1.084 - 3.464** |
| SARS-CoV-2 detection | **0.034ǂ** |  |  |  |  |
| Yes / No |  | 0.597 | 0.340 - 1.0049 | **0.508** | **0.269 - 0.956** |
|  |  |  |  |  |  |
|  |  | Cough | | | |
| Number of virus infection/coinfection | 0.313ǂ |  |  |  |  |
| 1 |  | 1.401 | 0.818 - 2.398 | 1.292 | 0.746 - 2.239 |
| 2 |  | 2.08 | 0.939 - 4.616 | 1.641 | 0.724 - 3.720 |
| 3 |  | **3.817** | **1.073 - 13.571** | 2.755 | 0.757 - 10.022 |
| Coinfection | 0.141ǂ |  |  |  |  |
| Yes / No |  | **2.063** | **1.071 - 3.971** | 1.637 | 0.832 - 3.221 |
| SARS-CoV-2 detection | **0.013ǂ** |  |  |  |  |
| Yes / No |  | **0.371** | **0.207 - 0.665** | **0.449** | **0.240 - 0.840** |
|  |  |  |  |  |  |
|  |  | Headache | | | |
| Number of virus infection/coinfection | - | - | - | - | - |
| 1 |  | - | - | - | - |
| 2 |  | - | - | - | - |
| 3 |  | - | - | - | - |
| Coinfection | 0.481ǂ |  |  |  |  |
| Yes / No |  | 0.251 | 0.032 - 1.955 | 0.481 | 0.053 - 4.317 |
| SARS-CoV-2 detection | 0.558ǂ |  |  |  |  |
| Yes / No |  | 1.239 | 0.334 - 4.585 | 0.674 | 0.173 - 2.624 |
|  |  |  |  |  |  |
|  |  | Coryza | | | |
| Number of virus infection/coinfection | 0.117ǂ |  |  |  |  |
| 1 |  | 1.668 | 0.975 - 2.854 | 1.468 | 0.828 - 2.602 |
| 2 |  | **3.728** | **1.850 - 7.513** | **2.371** | **1.117 - 5.032** |
| 3 |  | **2.925** | **1.201 - 7.118** | 2.117 | 0.806 - 5.559 |
| Coinfection | **0.043ǂ** |  |  |  |  |
| Yes / No |  | **2.545** | **1.505 - 4.303** | **1.804** | **1.016 - 3.203** |
| SARS-CoV-2 detection | 0.274ǂ |  |  |  |  |
| Yes / No |  | **0.422** | **0.221 - 0.806** | 0.673 | 0..329 - 1.378 |
|  |  |  |  |  |  |
|  |  | Nausea | | | |
| Number of virus infection/coinfection | - | - | - | - | - |
| 1 |  | - | - | - | - |
| 2 |  | - | - | - | - |
| 3 |  | - | - | - | - |
| Coinfection | 0.686ǂ |  |  |  |  |
| Yes / No |  | 0.548 | 0.157 - 1.915 | 0.771 | 0.211 - 2.809 |
| SARS-CoV-2 detection | 0.097ǂ |  |  |  |  |
| Yes / No |  | **3.041** | **1.200 - 1.706** | 2.347 | 0.887 - 6.210 |
|  |  |  |  |  |  |
|  |  | Sore throat | | | |
| Number of virus infection/coinfection | 0.892ǂ |  |  |  |  |
| 1 |  | 0.755 | 0.289 - 1.971 | 0.718 | 0.257 - 2.003 |
| 2 |  | 0.723 | 0.187 - 2.794 | 0.649 | 0.151 - 2.777 |
| 3 |  | 0.472 | 0.057 - 3.907 | 0.567 | 0.061 - 5.212 |
| Coinfection | 0.648ǂ |  |  |  |  |
| Yes / No |  | 0.741 | 0.243 - 2.26 | 0.758 | 0.227 - 2.532 |
| SARS-CoV-2 detection | 0.956ǂ |  |  |  |  |
| Yes / No |  | 1 | 0.325 - 3.069 | 0.965 | 0.272 - 3.422 |
|  |  |  |  |  |  |
|  |  | Diarrhea | | | |
| Number of virus infection/coinfection | 0.522ǂ |  |  |  |  |
| 1 |  | 2.117 | 0.548 - 8.174 | 2.517 | 0.220 - 0.575 |
| 2 |  | 3.130 | 0.673 - 14.546 | 3.101 | 0.195 - 0.559 |
| 3 |  | 1.5 | 0.149 - 15.051 | 1.773 | 0.651 - 0.148 |
| Coinfection | 0.502ǂ |  |  |  |  |
| Yes / No |  | 1.584 | 0.532 - 4.712 | 1.479 | 0.482 - 4.538 |
| SARS-CoV-2 detection | 0.107ǂ |  |  |  |  |
| Yes / No |  | 2.140 | 0.715 - 6.407 | 3.017 | 0.833 - 10.916 |
|  |  |  |  |  |  |
|  |  | Vomit | | | |
| Number of virus infection/coinfection | 0.868ǂ |  |  |  |  |
| 1 |  | 1.013 | 0.501 - 2.048 | 0.997 | 0.489 - 2.034 |
| 2 |  | 1.674 | 0.713 - 3.931 | 1.387 | 0.584 - 3.297 |
| 3 |  | 1.484 | 0.487 - 4.521 | 1.184 | 0.384 - 3.646 |
| Coinfection | 0.419ǂ |  |  |  |  |
| Yes / No |  | 1.597 | 0.831 - 3.070 | 1.318 | 0.678 - 2.562 |
| SARS-CoV-2 detection | 0.872ǂ |  |  |  |  |
| Yes / No |  | 0.643 | 0.274 - 1.504 | 1.078 | 0.430 - 2.704 |
|  |  |  |  |  |  |
|  |  | Hospitalization time (days) | | | |
| Number of virus infection/coinfection ^b^ | - |  |  |  |  |
| 1 |  | 1.975 | -3.149 - 7.099 | 4.127 | -0.853 - 9.108 |
| 2 |  | -6.728 | -13.638 - 0.182 | -2.159 | -9.058 - 4.740 |
| 3 |  | -7.908 | -16.890 - 1.073 | 1.282 | -7.802 - 10.368 |
| Coinfection | - |  |  |  |  |
| Yes / No |  | **-8.236** | **-13.555 - -2.918** | -3.339 | -8.773 - 2.093 |
| SARS-CoV-2 detection | - |  |  |  |  |
| Yes / No |  | 14.135 | 8.480 - 19.790 | 10.151 | 4.372 - 15.931 |
|  |  |  |  |  |  |
|  |  | ICU | | | |
| Number of virus infection/coinfection | 0.999ǂ |  |  |  |  |
| 1 |  | 0.944 | 0.471 - 1.888 | 0.999 | 0.489 - 2.042 |
| 2 |  | 0.900 | 0.347 - 2.330 | 1.062 | 0.395 - 2.856 |
| 3 |  | 0.754 | 0.202 - 2.800 | 0.981 | 0.253 - 3.805 |
| Coinfection | 0.926ǂ |  |  |  |  |
| Yes / No |  | 0.878 | 0.415 - 1.859 | 1.037 | 0.471 - 2.284 |
| SARS-CoV-2 detection | 0.878ǂ |  |  |  |  |
| Yes / No |  | 1.221 | 0.570 - 2.612 | 1.066 | 0.470 - 2.419 |
|  |  |  |  |  |  |
|  |  | Supplemental oxygen | | | |
| Number of virus infection/coinfection | 0.489ǂ |  |  |  |  |
| 1 |  | 1.004 | 0.611 - 1.650 | 0.987 | 0.582 - 1.675 |
| 2 |  | 1.65 | 0.824 - 3.303 | 1.245 | 0.589 - 2.632 |
| 3 |  | **3.4** | **1.191 - 9.703** | 2.149 | 0.705 - 6.547 |
| Coinfection | 0.213ǂ |  |  |  |  |
| Yes / No |  | **2.042** | **1.172 - 3.557** | 1.461 | 0.799 - 2.671 |
| SARS-CoV-2 detection | 0.081ǂ |  |  |  |  |
| Yes / No |  | **0.411** | **0.232 - 0.728** | 0.577 | 0.311 - 1.072 |
|  |  |  |  |  |  |
|  |  | Mechanical ventilation | | | |
| Number of virus infection/coinfection | 0.310ǂ |  |  |  |  |
| 1 |  | 1.104 | 0.504 - 2.419 | 1.144 | 0.503 - 2.602 |
| 2 |  | 0.343 | 0.073 - 1.597 | 0.326 | 0.067 - 1.592 |
| 3 |  | 1.125 | 0.292 - 4.325 | 1.238 | 0.291 - 5.262 |
| Coinfection | 0.259ǂ |  |  |  |  |
| Yes / No |  | 0.556 | 0.207 - 1.492 | 0.571 | 0.206 - 1.583 |
| SARS-CoV-2 detection | 0.996ǂ |  |  |  |  |
| Yes / No |  | 1.188 | 0.491 - 2.873 | 1.002 | 0.401 - 2.499 |
|  |  |  |  |  |  |
|  |  | Death | | | |
| Number of virus infection/coinfection | - | - | - | - | - |
| 1 |  | - | - | - | - |
| 2 |  | - | - | - | - |
| 3 |  | - | - | - | - |
| Coinfection | 0.618ǂ |  |  |  |  |
| Yes / No |  | 0.340 | 0.077 - 1.495 | 1.610 | 0.257 - 10.077 |
| SARS-CoV-2 detection | 0.490ǂ |  |  |  |  |
| Yes / No |  | 1.443 | 0.507 - 4.106 | 0.653 | 0.190 - 2.241 |

^a^ Logistic regression with adjustment for confounders: age (all age groups), sex, comorbidities (hypertension, smoker, cardiovascular disease, neoplasm, diabetes, asthma, neurological disease, obesity, COPD, dyslipidemia, transplants, kidney disease, others), COVID-19 vaccination status (no vaccinated, partially, complete)

^b^ Linear regression with adjustment for confounders: age (all age groups), sex, comorbidities (hypertension, smoker, cardiovascular disease, neoplasm, diabetes, asthma, neurological disease, obesity, COPD, dyslipidemia, transplants, kidney disease, others), COVID-19 vaccination status (no vaccinated, partially, complete)

Empty rows: mental confusion: no patient with 2 and ≥3 coinfections; Headache: no patient with 2 coinfections; Nausea: no patient with ≥3 coinfections; Death: no patient with ≥3 coinfections.

ǂ p-value to the likelihood ratio test
